# Combined Lumbar-Sacral Plexus Block in Facioscapulohumeral Muscular Dystrophy for Hip Fracture Surgery: A Case Report

**DOI:** 10.4274/TJAR.2024.231471

**Published:** 2024-02-28

**Authors:** Mete Manici, İlayda Kalyoncu, Cemil Cihad Gedik, Mehmet Ali Deveci, Yavuz Gürkan

**Affiliations:** 1 Koç University Faculty of Medicine, Department of Anaesthesiology and Reanimation, İstanbul, Turkey; 2 Koç University Faculty of Medicine, Department of Orthopaedic Surgery, İstanbul, Turkey

**Keywords:** Facioscapulohumeral muscular dystrophy, hip fracture, lumbosacral plexus block, muscular dystrophy, regional anaesthesia

## Abstract

Facioscapulohumeral muscular dystrophy (FSHD) is a muscular dystrophy that can affect individuals of all age groups. Its prevalence is reported to be 0.4-1 in 10,000 people. Because of the low occurrence of FSHD, anaesthetic management is primarily based on expert opinions, case reviews, or brief series. Here, we present the case of a 72-year-old woman with FSHD who underwent hip fracture (HF) surgery. To prevent respiratory compromise due to FSHD, we opted for lumbar-sacral plexus block. To the best of our knowledge, there is no information in the literature regarding the use of combined lumbar-sacral plexus block in patients with FSHD undergoing HF surgery.

**Main Points**

• Facioscapulohumeral muscular dystrophy (FSHD) is a muscular dystrophy that affects individuals of all age groups. Its prevalence is reported to be 0.4 to 1 in 10,000 people.

• There is no established guiding principle for anesthetic management in FSHD. Based on the limited number of published case reviews, it is advisable to avoid triggers for malignant hyperthermia, and neuromuscular blockade should be used cautiously.

• We aimed to share our anesthesia experience for HF in a geriatric patient with FSHD respiratory compromise.

## Introduction

Facioscapulohumeral muscular dystrophy (FSHD) is a muscular dystrophy that affects individuals of all age groups. Its prevalence is reported to be 0.4-1 in 10,000 people.^1^ The typical presentation of FSHD includes selective weakness of the shoulder girdle and facial muscles.^2^ Common complications include pulmonary dysfunction, cardiac abnormalities, muscle contracture, and susceptibility to malignant hyperthermia.^3^

There is no established guiding principle for anaesthetic management in FSHD. On the basis of the limited number of published case reviews, it is advisable to avoid triggers for malignant hyperthermia, and neuromuscular blockade should be used cautiously.^4^

Anaesthesia management for hip fracture (HF) can be provided with either general or regional anaesthesia. Peripheral nerve blocks are other options for providing anaesthesia for elderly and high-risk patients. Combined lumbar-sacral plexus block (CLSB) has been introduced as a peripheral regional anaesthesia method in HF surgery.^5,6^

We shared our anaesthesia experience with HF in a geriatric patient with FSHD respiratory compromise.

## Case Presentation

A 72-year-old female, weighing 70 kg, with a medical history of FSHD and hypertension, classified as American Society of Anesthesiologists Physical Status III, was scheduled for left HF surgery for intertrochanteric fracture repair. The patient was diagnosed with severe FSHD at the age of 45 years, experiencing respiratory muscle weakness necessitating bilevel positive airway pressure ventilation and severe muscle wasting.

In 2019, the patient underwent an appendectomy under general anaesthesia, after which she had an extended stay in the intensive care unit (ICU) and required mechanical ventilation support. The patient refused to receive general anaesthesia because of this experience. Central neuraxial blocks were not technically possible because of previous scoliosis surgery. Consequently, CLSB was planned. Written informed consent was obtained from the patient for the publication of this report.

In the operating room, vital signs were monitored. Oxygen supplement via nasal cannula flow (2-3 L h) was given to the patient. Midazolam (1 mg) and fentanyl (25 µg) were intravenously administered before positioning to make the patient more comfortable.

The patient was placed in the lateral decubitus position, with the operated side on the upper side. We performed all peripheral nerve blocks under ultrasound guidance (GE Logic P9, Gyeonggi-do, Republic of Korea), combined with a nerve stimulator (Stimuplex DIG/HNS11; B. Braun, Melsungen, Germany), and selected a 10 cm 22G needle (BBraun, Melsungen, Germany).

**Figure 1:**
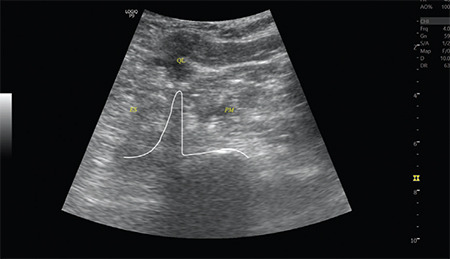
US anatomy of the left lumbar region at the level of the L3 transverse process in the shamrock view. The shamrock view with three leaves consists of the erector spinae (ES), quadratus lumborum (QL), and psoas major muscles (PM) and the transverse process of the L3 vertebral body (VB).

Lumbar plexus block was performed using the Shamrock approach.^7^ The kidney, psoas major, quadratus lumborum, erector spinae muscles, transverse/spinous processes, and vertebral body were visualized (Figure 1). Contact between the needle tip and the transverse process during needle insertion was avoided by slightly tilting the probe caudad until the transverse process disappeared and the bulging edge of the vertebral body together with the psoas muscle was visualized. Using a nerve stimulator, an appropriate needle position was adjusted and confirmed as having a quadriceps contraction with a stimulating current of 0.5 mA. Following this, 15 mL 2% lidocaine, 10 mL 0.5% bupivacaine, and 5 mL 0.9% NaCl were injected.

**Figure 2:**
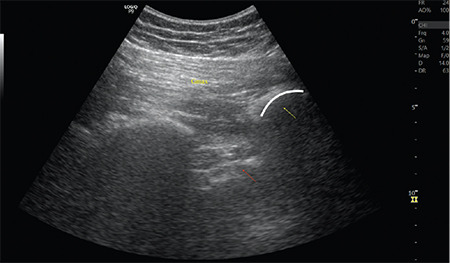
Gluteus maximus muscle (Gmax), sacral bone (yellow arrow), and hyperechoic sacral plexus (red arrow).

In the same position, an ultrasound-guided parasacral parallel shift approach was performed for sciatic nerve block as described by Bendtsen et al.^8^ The transducer is aligned between the posterior superior iliac spine and the midpoint of the line connecting the posterior superior iliac and the greater trochanter and the iliac bone line identified. The transducer is moved inferomedially with a parallel parasacral shift. When the transducer beam arrives at the sciatic notch, the ultrasonographic continuity of the iliac bone line is interrupted. This is exactly where the sacral plexus exits the pelvis (Figure 2). A 0.5 mA stimulating current was used to verify the sciatic nerve by observing foot plantar flexion or dorsiflexion. Following this, 10 mL of 2% lidocaine and 10 mL of 0.5% bupivacaine were injected.

After the block performances, the patient was placed in the supine position. Thirty minutes after the block performance, sensory block testing on ice was used, and surgery was allowed to start. At the beginning of surgery, she had some pain; therefore, an additional 25 µg fentanyl intravenous (IV) was administered. Subsequently, she did not express any pain at any point during the procedure.

The procedure lasted 55 min. The patient stayed in the intensive care unit one night for a close follow-up. The patient needed NIMV support at night. Fentanyl patient-controlled analgesia IV (no background infusion, 10 µg bolus dose, 10-minute lock-out time) was administered to the patient. Additionally, she received 3x1 g acetaminophen. The postoperative visual analogue scale scores ranged from 0 to 1. During the first 24 h postoperatively, total fentanyl consumption was 20 µg. The patient did not have any respiratory compromise postoperatively. There were no postoperative complications. On the fifth day following surgery, the patient was discharged uneventfully.

## Discussion

FSHD may present with a variety of symptoms, and its progression is also variable. Extramuscular manifestations of FSHD are rare. Clinically significant respiratory insufficiency occurs in 1% of patients with FSHD.^9,10^ Nevertheless, special attention is necessary. Regional anaesthesia techniques may be a suitable choice for anaesthesia management in FSHD.

The patient refused general anaesthesia because of a previous long ICU stay after appendectomy surgery under general anaesthesia. To avoid respiratory failure, malignant hyperthermia, and use of neuromuscular blockade, we preferred the regional anaesthesia technique in this case.

The patient underwent scoliosis surgery with T2 iliac wing posterior stabilization and fusion with L3-L5 and Th4-Th6 vertebroplasty. Whether corrected or uncorrected, the anatomical anomalies of scoliosis can hinder the placement and effectiveness of neuraxial anaesthesia.^11^ Neuroaxial blocks were not technically possible; therefore, we did not consider them as a first-line choice.

Therefore, we performed lumbar and sacral plexus nerve block combination. In the beginning, the patient had some pain, which was relieved by the addition of 25 µg of fentanyl. Because of the lack of sensory block of T12, partial failures could be observed in the lumbar and sacral plexus nerve block combination. A low- dose systemic analgesic may be required in these cases. Following this, all procedures were completed without pain. We did not encounter any hemodynamic disturbance during surgery. The patient was comfortable breathing spontaneously with oxygen supplement via nasal cannula flow of 2-3 L h.

## Conclusion

The combination of lumbar and sacral plexus block may offer reliable surgical anaesthesia, adequate postoperative pain control, and reduced risk of HF surgery complications in geriatric patients with FSHD-related respiratory compromise.
